# Effects of continuity of care on hospital admission in patients with type 2 diabetes: analysis of nationwide insurance data

**DOI:** 10.1186/s12913-015-0745-z

**Published:** 2015-03-17

**Authors:** Kyoung Hee Cho, Sang Gyu Lee, Byungyool Jun, Bo-Young Jung, Jae-Hyun Kim, Eun-Cheol Park

**Affiliations:** Department of Public Health, Graduate School, Yonsei University, Seoul, South Korea; Institute of Health Services Research, College of Medicine, Yonsei University, Seoul, South Korea; Graduate School of Public Health, Yonsei University, Seoul, South Korea; Health Insurance Review & Assessment Institute, Health Insurance Review & Assessment Service, Seoul, South Korea; Department of Preventive Medicine and Institute of Health Services Research, Yonsei University College of Medicine, 50 Yonsei-ro, Seodaemun-gu, Seoul, 120-752 South Korea

**Keywords:** COC, Continuity of care, ICOC, SECON, Type 2 diabetes, UPC

## Abstract

**Background:**

A system for managing chronic disease including diabetes mellitus based on primary care clinics has been used in Korea since April 2012. This system can reduce copayments for patients that are managed by a single primary-care provider and lead to improve continuity of care. The aim of this study is to determine whether there is an association between continuity of care for outpatients and hospital admission and identify the continuity index that best explains hospital admissions for patients with type 2 diabetes.

**Methods:**

We performed a cross-sectional study using 2009 National Health Insurance Sample (NHIS) from the Health Insurance Review & Assessment Services (HIRA) of Korea. The dependent variable was hospital admission due to type 2 diabetes mellitus. Continuity of care was measured using the Usual Provider Care index (UPC), Continuity of Care index (COC), Sequential Continuity of Care index (SECON), and Integrated Continuity of Care index (ICOC).

**Results:**

Patients with low COC scores (<0.75) were more likely to be hospitalized [odds ratio, 2.44; 95% CI, 2.17–2.75] compared with the reference group (COC ≥0.75), after adjusting for all covariates. we calculated the area under the receiver operating characteristic (AUROC) curve for each index to find which index had the greatest explanatory ability for hospital admission. The AUROC of the COC was the greatest (0.598), but the AUROC curves for the UPC (0.597), SECON (0.593), and ICOC (0.597) were similar.

**Conclusions:**

High continuity of care may reduce the likelihood for hospital admission. The COC had marginally more explanatory power.

## Background

A system for managing chronic disease including diabetes mellitus based on primary care clinics has been used in Korea since April 2012. This system can reduce copayments for patients that are managed by a single primary-care provider [1].

The prevalence of diabetes mellitus has increased from approximately 2% in the 1970s to more than 10% in the early 1990s and 10.5% in 2011 [2]. Furthermore, the proportion of medical costs due to diabetes among total health care expenditures is increasing [3], and the disease burden is expected to be much greater in the future due to increases in life expectancy [4,5], a westernized lifestyle, and an aging society [6]. Diabetes mellitus was the fifth leading cause of death according to the National Statistics Office in 2012 [7]. Also heart diseases and cerebrovascular diseases, the second and third leading causes of death, are likely to be influenced by diabetes, considering that diabetes increases the mortality rate twofold to fourfold in patients with cardiovascular diseases and stroke [8]. Although diabetic complications or disabilities can be effectively reduced by periodic monitoring, dietary modification, medication, and early intervention for complications [9,10], the hospitalization rate for patients with uncontrolled diabetes in Korea was 127.6 people per 100,000 people in the population, more than two times the OECD (Organization for Economic Cooperation and Development) average of 50.3 people per 100,000 people in the population in 2011 [11].

In order to efficiently manage chronic diseases with limited resources, policymakers are becoming interested in the benefits of continuity of care, which can reduce the risks of diabetic complications [12], improve preventive care [13,14], increase patient satisfaction [15] and compliance [16,17], and decrease emergency and inpatient medical services and care costs [18-20]. Previous studies have shown that fragmented visit patterns [21-24], a shortage of primary care [25], and difficulty in accessing ambulatory care are related to preventable hospitalization [26,27]. In Korea, the National Health Insurance based on universal coverage has improved accessibility to medical care, and geographical accessibility are better because Korea is limited in area and has better transportation between regions. As accessibility of medical care improves, the causes of preventable hospitalization seem to be due to pattern of health care utilization. Also changes in health care management including shifts toward multidisciplinary group practices can be lead to fragmented visit patterns. Especially, Korea’s system is quite different from the managed care delivery system of the US where a patient’s selection of health care provider is regulated and restricted [28]. In Korea, primary care physicians work mostly in solo private practices and are reimbursed on a fee-for-service basis. This system enables patients to choose and retain an individual physician regardless of changes in employment status. It is important to evaluate the consequences of fragmented care visits through empirical study.

Although continuity of care is one of the desirable attributes that define primary care [29,30], empirical studies of associations between continuity of care and health outcomes in Korea are rare. Although continuity of care can be measured differently according to how it is defined, few studies have addressed this point.

The aims of this study were to analyze the association between continuity of ambulatory care and hospital admission and determine which continuity index has the best explanatory ability for hospital admission among patients with type 2 diabetes mellitus.

## Methods

### Data source

This study used data from the 2009 National Health Insurance Sample (NHIS) of the Health Insurance Review & Assessment Services (HIRA) of Korea. The NHIS includes 1,100,000 patients who can represent the country as a whole (46,000,000 people) and is stratified according to sex, age in 5-year intervals, and hospital admission status. The 2009 data provided information from healthcare claims for about 1,100,000 patients, including all medical history during 2009, age, sex, costs, prescription history, diagnostic tests, and other factors. We used sampling weight to estimate the population. Ethical approval for this study was granted by the institutional review board of the Graduate School of Public Health, Yonsei University, Seoul, Korea.

### Study design

We performed a cross-sectional study to identify the association between the continuity of ambulatory care and hospital admission. We classified the patients into two groups based on hospital admission status and examined the factors that influenced hospital admission. We measured the continuity of care by four indices and investigated which index had the greatest explanatory ability for hospital admission among patients with type 2 diabetes mellitus.

### Study subjects

**Figure 1:**
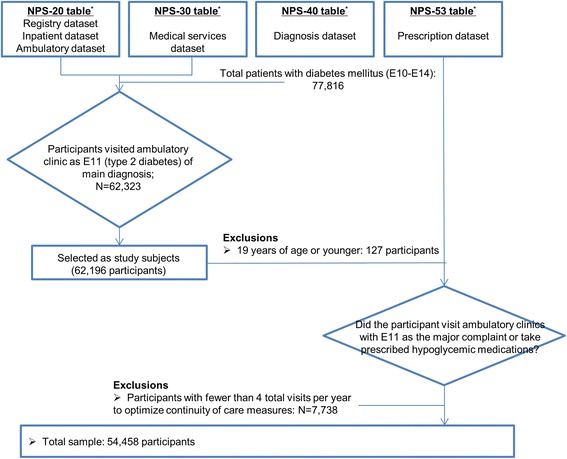
* The National Health Insurance Sample data consisted of 4 tables (20, 30, 40, and 53 table). Each table includes different information.

This study included only patients with type 2 diabetes, because the pathogenesis is different in type 1 and type 2 diabetes. We selected patients who visited outpatient clinics and were given a major diagnosis code of E11, indicating type 2 diabetes based on the International Classification of Diseases-10. We defined medical usage as cases in which patients visited an outpatient clinic and received a main diagnosis of E11 or were prescribed hypoglycemic agents. The total number of patients with diabetes was 77,816 and comprised 3,234 with type 1 diabetes, 62,323 with type 2 diabetes, and 12,259 patients with the other types of diabetes such as diabetes related with nutrition deficiency. From 62,323 patients with type 2 diabetes, we excluded 127 patients with type 2 diabetes who were less than 20 years old and 7,738 patients whit less than four outpatient visits during the year. This criterion was intended to facilitate calculation of the continuity of care index in a structurally reasonable and meaningful manner. Another rationale for this criterion was to improve the accuracy of type 2 diabetes diagnosis. We obtained diabetes diagnoses from data, which are based on information from the HIRA claims database. The accuracy of diagnosis in HIRA claims data is roughly 70% [31]. Thus, the final study population included 54,458 patients (Figure 1).

### Measures of study variables

#### Outcome variable

The dependent variable in this study was hospital admission due to type 2 diabetes mellitus, defined by the usage of inpatient medical services for more than 1 day and a main diagnosis code of E11.

#### Measurement of continuity of care

We measured continuity of care using the Usual Provider of Care (UPC), Continuity of Care (COC), Sequential Continuity of Care (SECON), and Integrated Continuity of Care (ICOC). The UPC, COC, and SECON are commonly used in healthcare practice. The ICOC was calculated by merging the UPC, COC, and SECON via a Principal Component Analysis (PCA), which used the weighted means of the variables for each type of index. The index values ranged from 0 to 1, with higher values indicating a higher continuity of care. Each index highlights a different aspect of continuity of care. The UPC highlights the density of care from the usual provider and measures the frequency of a patient’s visits to a physician, dividing the visits to the same physician by the total number of visits during the defined time period [32]. The formula for the UPC is as follows:

1 $$ UPC=\frac{\overset{max}{1\le }j\le M(nj)}{N} $$

The COC emphasizes the distribution of visits to each healthcare provider that the patient visited [33]. One of the advantages of this index is that it reflects coordination of care arising when one provider refers a patient to another provider who takes the patient referral again. The formula for the COC is as follows:

2 $$ COC=\frac{{\displaystyle \sum_{j=1}^M{n_j}^2-N}}{N\left(N-1\right)} $$

The SECON emphasizes the sequence of care from the same provider. This index sequentially measures the various physicians visited [34]. The formula for the SECON is as follows:

3 $$ SECON=\frac{{\displaystyle \sum_{i=1}^{N-1}Si}}{N-1} $$

- ◼ N: total number of visits(only ambulatory visits)
- ◼ *n*_*j* :_ number of visits to the *j*th different provider*, j* = 1,2…M
- ◼ M: number of potentially available providers; ‘potentially available providers’ means providers which patient visited in this study.
- ◼ *Si*: if the visit and the subsequent (*i* + 1)th visit are to the same provider then *Si* = 1, and *Si* = 0 otherwise.

The ICOC integrates the other three indices to give a different perspective when measuring continuity of care [35]. The general formula for the ICOC index is as follows:

4 $$ ICOC=\left({\ss}_1UPC + {\ss}_2COC + {\ss}_3 SECON\right)/\left({\ss}_1+{\ss}_2 + {\ss}_3\right) $$

where *ß*_*1*_*,ß*_*2*_*,* and *ß*_*3*_ is the first principal component Eigenvector of the PCA result. We used the COC to identify the relationship between the continuity of ambulatory care and hospital admission status. In this analysis, high continuity was defined a COC value of 0.75 or more [36]. To find which index has the greatest explanatory ability for hospital admission, we divided the scores for the four indices into five categories: 1.00 (perfect), 0.76–0.99 (very high), 0.51–0.75 (high), 0.26–0.50 (low), and 0.00–0.25 (very low). We then identified the risks for hospital admission according to the continuity level and identified the index with the greatest explanatory ability.

### Other covariates

The covariates included age, sex, health insurance type, severity of disease, number of combination hypoglycemic agents, main attending clinic, type of diabetic medication prescribed, number of tests performed, and medication prescription days. In case of health insurance, people can qualify for medical aids whose household income is less than $ 600 per month based on single household. We calculated two values to define the severity of disease and used the larger of the two values for our analysis. One value was the patient clinical complexity level using the Korean Diagnosis Related Groups (KDRG) code. The KDRG code accepted at face value the major diagnostic categories system of USA. The other value was the total number of diabetic complications based on diagnosis codes throughout all medical care during 2009. We used the three proxy variables for patient severity. If blood glucose level is not controlled, patients with type 2 diabetes are prescribed combination therapies with hypoglycemic agents. We judged that the number of prescribed combinations of hypoglycemic agents might reflect the real disease severity. The main attending clinic was the healthcare institution visited most frequently by the patient for outpatient care and if visit frequency per institution was same, the main attending clinic was the healthcare institution visited the latest. If the severity of disease is greater, patients in Korea use a higher-level hospital.

### Statistical analysis

We divided the patients into two groups based on whether experienced hospital admission or not and compared the differences of the distribution of each relevant factor between the groups using the chi-square test and used sampling weight to estimate population. The relationship between the continuity of care and hospital admission was evaluated by a multiple logistic regression analysis. To identify the association between continuity of care and hospital admission, we had to do only one index. We could not put four continuity indices in model at the same time because of multicollinearity among indices. So we selected COC. This index is the most commonly used and can reflect the number of total visits and the number of health care provider who patients visit. We classified stratified to scores of COC. We defined high as COC scores were more than 0.75, and low as COC scores were less than 0.75. We tested the goodness-of-fit of model using the Log Likelihood Ratio. We also tested the linear trend in odds ratios across the five categories of continuity level and calculated the area under the receiver operating characteristic (AUROC) curve for each continuity index in a multiple logistic regression. We performed a PCA to calculate ICOC scores and used Correlation Coefficients to compare the correlates between the ICOC and the other continuity indices to assess the explanatory ability of the ICOC and the correlations of the PCA results. Our analysis was performed using the SAS statistical software (version 9.2).

## Results

In our sample, 4.0% of the patients experienced hospital admission and 96.0% did not experience hospital admission. The two groups were significantly different in all of the individual patient characteristics (Table 1).

**Table 1:** **Distribution of individual patient characteristics by hospital admission status**

						Unit*: persons (%)
**Characteristics**	**Yes**		**No**		**Total**	**P-value**
**Age (years)**						
20–29	492	(6.8)	6,723	(93.2)	7,215	<.0001
30–39	1,977	(4.3)	44,169	(95.7)	46,146	
40–49	7,415	(3.7)	190,437	(96.3)	197,852	
50–59	11,885	(3.3)	348,114	(96.7)	359,998	
60–69	13,715	(3.3)	402,021	(96.7)	415,736	
≥70	19,308	(5.6)	323,871	(94.4)	343,179	
**Sex**						
Male	25,861	(3.6)	690,913	(96.4)	716,774	<.0001
Female	28,931	(4.4)	624,422	(95.6)	653,353	
**Health insurance type**						
National health Insurance	43,077	(3.4)	1,209,520	(96.6)	1,252,597	<.0001
Medical Aid	11,262	(10.2)	98,692	(89.8)	109,953	
Others	453	(6.0)	7,123	(94.0)	7,577	
**Severity** ^**†**^						
0	9,654	(2.1)	446,796	(97.9)	456,450	<.0001
1	26,600	(3.8)	672,502	(96.2)	699,102	
2	15,438	(7.8)	182,160	(92.2)	197,598	
≥3	3,100	(18.3)	13,877	(81.7)	16,977	
**Number of combination hypoglycemic agents**						
No medication	27,131	(6.6)	386,204	(93.4)	413,335	<.0001
1 agent	13,192	(2.9)	438,165	(97.1)	451,358	
2 agents	10,977	(2.7)	393,227	(97.3)	404,204	
≥3 agents	3,492	(3.4)	97,738	(96.6)	101,230	
**Diabetic medication**						
No medication	27,131	(6.6)	386,204	(93.4)	413,335	
Oral	25815	(2.8)	907507	(97.2)	933,323	
Insulin injection or pump	1846	(7.9)	21623	(92.1)	23,469	
**Main attending clinic**						
General hospital	24,315	(7.1)	319,543	(92.9)	343,858	<.0001
Hospital	7,300	(7.7)	88,053	(92.3)	95,353	
Clinical	21,600	(2.5)	833,908	(97.5)	855,508	
Public health center	1,577	(2.1)	73,830	(97.9)	75,407	
**Number of had tested** ^**§**^						
None	13,454	(4.3)	300,036	(95.7)	313,489	.0035
1	35,615	(4.0)	864,939	(96.0)	900,554	
≥2	5,723	(3.7)	150,361	(96.3)	156,084	
**Medication Prescription Days**						
0 days & Severity = 0″	699	(1.9)	35,430	(98.1)	36,130	<.0001
0 days & Severity ≥1‴	2,277	(4.3)	50,523	(95.7)	52,799	
1–179	18,308	(8.4)	198,914	(91.6)	217,222	
180–269	10,969	(6.1)	169,360	(93.9)	180,330	
270–359	15,846	(3.4)	451,804	(96.6)	467,650	
≥360	6,692	(1.6)	409,304	(98.4)	415,996	
**Level of continuity**						
High (COC scores ≥0.75)	27,508	(2.7)	984,191	(97.3)	1,011,698	<.0001
Low (COC scores <0.75)	27,285	(7.6)	331,144	(92.4)	358,428	

*Weighted frequency (weighted percent).

^†^used larger value whether number of complications related to diabetes mellitus or PCCL (Patient Clinical Complexity Level) using KDRG code.

^§^Tests included: HbA1c test, Glucose test, Lipid profiles and Fundus examination (fundus examination, fundus photography and fluorescence fundus angiography);

″Patients did not need medications; ‴patients who need medication but did not take prescriptions.

The logistic regression analyses showed that the continuity of care was significantly related to hospitalization, with the odds ratio of hospital admission being low for patients who had good continuity of care (Table 2). The reference group for comparison was patients with a COC score of 0.75 or more. The unadjusted odds ratio for hospital admission was 2.95 [95% CI: 2.78–3.12], and after adjusting for all covariates including disease severity, the odds ratio for hospital admission was 2.44 [95% CI: 2.17–2.75].

**Table 2:** **Odds Ratios for hospital admission according to individual characteristics**

	**Unadjusted**		**Adjusted**	
**Characteristics**	**Odds ratio**	**95% CI**	**Odds ratio**	**95% CI**
**Age(years)**				
20–29	1.23	0.88–1.71	1.07	0.72–1.59
30–39	0.75	0.64–0.88	0.84	0.71–1.00
40–49	0.65	0.60–0.72	0.74	0.67–0.83
50–59	0.57	0.53–0.62	0.69	0.63–0.75
60–69	0.57	0.53–0.62	0.66	0.61–0.72
≥70	1.00	-	1.00	-
**Sex**				
Male	1.00	-	1.00	-
Female	1.24	1.17–1.31	1.11	1.04–1.19
**Health security**				
Health insurance	1.00	-	1.00	-
Medical aid	3.20	2.96–3.47	2.32	2.11–2.54
Others	1.79	1.29–2.48	0.91	0.63–1.30
**Severity**				
0	1.00	-	1.00	-
1	1.83	1.70–1.97	1.75	1.60–1.90
2	3.92	3.60–4.27	3.05	2.76–3.37
≥3	10.34	8.64–12.38	6.85	5.62–8.34
**Number of combination hypoglycemic agents**				
No medication	2.33	2.18–2.50	1.77	1.62–1.93
1 agent	1.00	-	1.00	-
2 agents	0.93	0.86–1.01	1.14	1.04–1.25
≥3agents	1.19	1.05–1.34	1.46	1.27–1.67
**Diabetic medication**				
Oral	1.00	-	1.00	-
Insulin injection or pump	3.00	2.52–3.58	1.82	1.48–2.23
**Main attending clinic**				
General hospital	1.18	1.06–1.31	1.16	1.03–1.31
Hospital	1.08	0.99–1.19	1.20	1.08–1.34
Clinical	1.00	-	1.00	-
Public health center				
**Number of had tested**	2.94	2.76–3.13	3.01	2.80–3.23
None	3.20	2.91–3.52	2.60	2.33–2.91
1	1.00	-	1.00	-
≥2	0.83	0.71–0.97	0.89	0.75–1.07
**Medication prescription days**				
0 days & Severity = 0	1.21	0.95–1.53	1.86	1.43–2.43
0 days & Severity ≥1	2.76	2.36–3.22	2.05	1.71–2.45
1–179	5.63	5.14–6.16	3.72	3.34–4.16
180–269	3.96	3.59–4.37	3.41	3.06–3.81
270–359	2.15	1.96–2.34	2.07	1.87–2.28
≥360	1.00	-	1.00	-
Level of continuity*				
High (COC scores ≥ 0.75)	1.00	-	1.00	-
Low (COC scores < 0.75)	2.95	2.78–3.12	2.44	2.17–2.75

*to identify the association between continuity of care and hospital admission, we had to do only one index. We could not put four continuity indices in this model at the same time because of multicollinearity among indices; high defined as COC scores were more than 0.75, and low defined as COC scores were less than o.75.

**Figure 2:**
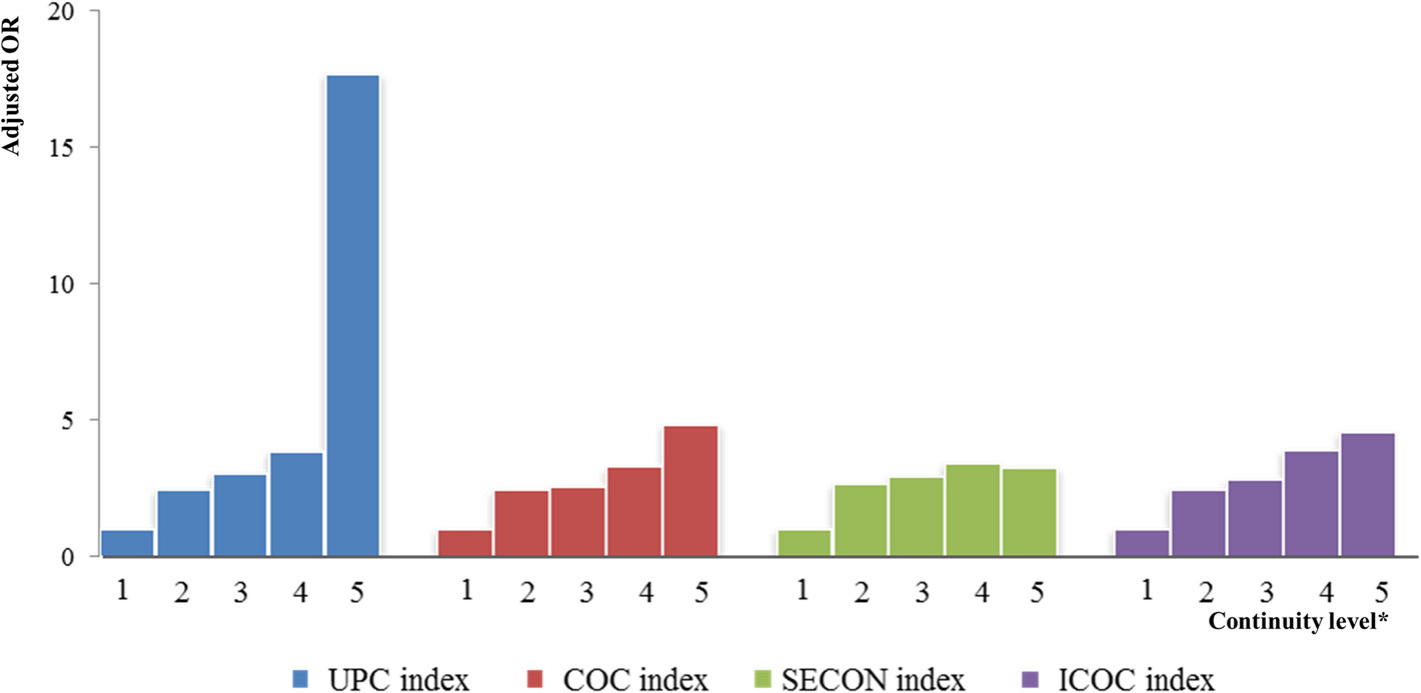
*1, Perfect 1.00; 2, 0.75–0.99; 3, 0.50–0.74; 4, 0.25–0.49; 5, 0.00–0.24.

Table 3 presents the distribution according to hospital admission and the relationship between the continuity level and hospital admission status for each continuity index. When compared with the reference group (patients with a continuity level of 1.00), the adjusted odds ratio of the group with a continuity level of 0.00-0.25 was 17.71 [95% CI: 8.51–36.86], 4.84[95% CI: 3.94–5.96], 3.27[95% CI: 2.53–4.23], and 4.60 [95% CI: 2.80–7.54] based on the UPC, COC, SECON, and ICOC, respectively. The unadjusted and adjusted odds ratios for hospital admission increased gradually as the level of continuity decreased based on all the continuity indices (p for trend: <.0001; Figure 2). Additionally, we calculated the AUROC curve for each index to find which index had the greatest explanatory ability for hospital admission. The AUC of the COC was the greatest (0.598), but the AUROC curves for the UPC (0.597), SECON (0.593), and ICOC (0.597) were similar.

**Table 3:** **Distribution and odds ratios for hospitalization by continuity index**

**Characteristics**	**Hospital admission**				**Total**	**P-value**	**Unadjusted OR**	**95% CI**	***P*** **for Trend** ^**†**^	**Adjusted OR** ^**‡**^	**95% CI**	***P*** **for trend** ^**†**^	**AUC** ^**§**^
	**Yes**	n (%)*	**No**	n (%)*									
**UPC**													
Perfect 1.0	21,108	(2.4)	866,469	(97.6)	887,576	<.0001	1.00	-	<.0001	1.00	-	<.0001	0.597
0.76–0.99	13,177	(5.7)	220,022	(94.3)	233,198		2.45	2.28–2.63		2.45	2.25–2.67		
0.51–0.75	14,231	(7.5)	175,545	(92.5)	189,776		3.39	3.15–3.64		3.09	2.84–3.36		
0.26–0.50	6,169	(10.4)	53,046	(89.6)	59,215		5.38	4.85–5.96		3.90	3.45–4.42		
0.00–0.25	107	(29.8)	253	(70.2)	362		14.09	11.72-16.94		17.71	8.51–36.86		
**COC**													
Perfect 1.00	21,108	(2.4)	866,469	(97.6)	887,576	<.0001	1.00	-	<.0001	1.00	-	<.0001	0.598
0.76–0.99	5,762	(5.1)	107,599	(94.9)	113,361		2.20	1.99–2.42		2.44	2.17–2.75		
0.51–0.75	11,262	(6.2)	169,976	(93.8)	181,237		2.72	2.52–2.94		2.58	2.36–2.83		
0.26–0.50	14,577	(8.4)	158,714	(91.6)	173,291		3.77	3.50–4.06		3.30	3.03–3.59		
0.00–0.25	2,085	(14.2)	12,577	(85.8)	14,661		6.81	5.66–8.19		4.84	3.94–5.96		
**SECON**													
Perfect 1.00	21,108	(2.4)	866,469	(97.6)	887,576	<.0001	1.00	-	<.0001	1.00	-	<.0001	0.593
0.76–0.99	15,215	(5.7)	253,183	(94.3)	268,398		2.47	2.30–2.65		2.70	2.49–2.93		
0.51–0.75	12,023	(8.1)	135,599	(91.9)	147,622		3.64	3.37–3.94		2.97	2.71–3.26		
0.26–0.50	5,262	(9.7)	48,961	(90.3)	54,223		4.41	3.95–4.93		3.43	3.03–3.88		
0.00–0.25	1,185	(9.6)	11,123	(90.4)	12,308		4.37	3.50–5.46		3.27	2.53–4.23		
**ICOC**													
Perfect 1.00	21,108	(2.4)	866,469	(97.6)	887,576	<.0001	1.00	-	<.0001	1.00	-	<.0001	0.597
0.76–0.99	9,715	(5.4)	170,937	(94.6)	180,652		2.33	2.15–2.53		2.49	2.26–2.74		
0.51–0.75	17,015	(7.1)	221,398	(92.9)	238,414		3.16	2.95–3.38		2.88	2.66–3.11		
0.26–0.50	6,508	(10.8)	53,930	(89.2)	60,438		4.95	4.46–5.50		3.92	3.48–4.42		
0.00–0.25	446	(14.6)	2,600	(85.4)	3,046		7.05	4.70–10.56		4.60	2.80–7.54		

UPC, usual provider care; COC, continuity of care; SECON, sequential continuity; ICOC, integrated continuity of care, AUC, area under the receiver operating characteristic curve).

*weighted frequency (weighted percent).

^†^ *P* for trend: wald Chi-square.

^‡^Odds Ratio are adjusted by each continuity index (UPC, COC, SECON, and ICOC) separately and all other independent variables because of multicollinearity between index.

^§^means discrimination ability of prediction model; The AUC of this model was 0.715.

## Discussion

Our findings indicate that high continuity of care reduced the odds ratio for hospital admission. Indeed, all four continuity indices displayed a trend for the odds ratio for hospital admission to decrease gradually as the continuity level increased.

The association between the continuity of care and hospital admission for the patients with type 2 diabetes in this study is consistent with previous studies of the association between the continuity of care and outcomes for patients with chronic conditions. Our study did not reveal the mechanism leading to these results. Gray et al. conducted a study of the theory of continuity of care, however, and suggested that continuity of care could provide benefits to patients by reinforcing the accumulation of patient knowledge, improving interpersonal communication, and increasing the likelihood that patients adhere to their doctors’ advice [37]. Another study found that the benefits of continuity may be magnified among patients with chronic disease, because patients with chronic disease are more likely than healthy persons to use outpatient services and may establish relationships with their physicians more quickly [38]. A third study suggested that a diabetes care model that integrates primary and specialty care, together with practices that adhere to guideline recommendations, was associated with a reduction in all-cause mortality and all types of hospitalizations (not only diabetes related), as compared with less structured models, without increasing direct health costs [39]. In Korea, more than $925 millions (US1$ = 1,000 Korean Won) were spent as a medical care spending due to diabetes in 2013, and of these 17.5% was attributable to preventable hospitalization such as hospital admission due to short or long term complication and due to uncontrolled diabetes mellitus [3]. If diabetes care model is reformed so primary care and specialty care are integrated, the preventable hospitalization could be reduced.

We evaluated four commonly used continuity indices to determine which index best explains hospital admissions for patients with type 2 diabetes. The AUROC curve of the COC was the largest, but the differences in explanatory ability among the indices were small. Hence, it may make little difference which index is used to measure the continuity of care for patients with type 2 diabetes. The definition of continuity is different for each index, however. Therefore, an index should be selected according to the features of the patients’ diseases and the policy related with the diseases, because the usage pattern for medical services will be different based on the policy.

This study has some limitations. The first limitation is the accuracy of the diagnosis. We used the NPS data based on the HIRA claims database. The accuracy of diagnosis in the KNHI claims data is about 70%. Therefore, when defining ambulatory care usage due to type 2 diabetes, we reviewed not only the diagnosis code but also the general components of the medications prescribed. Thus, we included taking prescriptions for hypoglycemic agents as medical usage for type 2 diabetes in case where the main diagnosis was not diabetes mellitus. Despite our efforts, the accuracy of diagnosis of type 2 diabetes in this study may be challenged. The second limitation is that we could not consider all the factors affecting the continuity of care and hospital admission, such as income level, education, residence area, health behaviors, and diagnosis date, because of the limitations of the claims data. Our analysis of income, for example, was limited to a dichotomous classification based on the type of health insurance. Furthermore, health behaviors and blood glucose control are highly related, but we could not identify the relationship directly. The third limitation is that our results could not reflect the characteristics of patients with fewer than four ambulatory visits. We excluded patients with fewer than four outpatient visits to increase the validity of our measurements of continuity. For example, in some cases where the actual continuity was not good, the continuity value was relatively high because the patient visited the same physician twice in the year. The fourth limitation is that we could not identify the individual service provider on the basis of the information in the claims database. Hence, the outpatient healthcare provider was not a physician but was instead a medical institution. The final limitation is that although we made an effort to restrict the variables to factors associated with type 2 diabetes, some of the tests performed at the ambulatory care clinics could have been for patients without diabetic complications. Because the study design was cross sectional, the problem of interpretative confusion may be raised in relation to the reasons for hospital admission.

Despite the limitations, our study has several strengths. First, we analyzed a representative sample of patients with type 2 diabetes mellitus using nationwide claims data. Second, unlike most previous studies, which adjusted for patient severity using only Charlsons’ comorbidity index, the number of comorbidities, and the number of diabetic complications, we adjusted for severity by dividing disease severity and patient severity. We also went to great lengths to adjust for severity using proxy variables that reflect real disease severity, such as the number and types of combinations of hypoglycemic agents.

In conclusion, our results support the hypothesis that reducing fragmented care and improving continuity of care can decrease hospital admissions for patients with chronic diseases including diabetes. We would encourage policymakers to recognize the need for an effective healthcare delivery system that promotes continuity of care, encouraging a team approach and enhancing accessibility.

## Conclusion

The likelihood of hospital admission for patients with type 2 diabetes declined gradually with increasing continuity of care. There were only small differences among the explanatory abilities of the four continuity indices. We measured continuity of care over a single year and analyzed the relation between the continuity of care and hospital admission. Further research is needed on the associations between long-term continuity of care and various healthcare outcomes.

## Abbreviations

HIRA: Health Insurance Review & Assessment Services
NHIS: National Health Insurance Sample
UPC: Usual Provider Care
COC: Continuity of Care
SECON: Sequential Continuity of Care
ICOC: Integrated Continuity of Care
PCA: Principal Component Analysis
AUROC: Area Under the Receiver Operating Curve
